# Investigation of the Trend of Selecting Anti-Vascular Endothelial Growth Factor Agents for the Initial Treatment of Neovascular Age-Related Macular Degeneration and Polypoidal Choroidal Vasculopathy

**DOI:** 10.3390/jcm10163580

**Published:** 2021-08-14

**Authors:** Jae-Hui Kim, Jong-Woo Kim, Chul-Gu Kim

**Affiliations:** Department of Ophthalmology, Kim’s Eye Hospital, Konyang University College of Medicine, Seoul 07301, Korea; kjwood@kimeye.com (J.-W.K.); chulgukim@kimeye.com (C.-G.K.)

**Keywords:** age-related macular degeneration, polypoidal choroidal vasculopathy, type 3 macular neovascularization, ranibizumab, aflibercept

## Abstract

BACKGROUND: This study aimed to investigate the trend of selecting ranibizumab and aflibercept for the initial treatment of neovascular age-related macular degeneration (AMD) and polypoidal choroidal vasculopathy (PCV). METHODS: This was a retrospective study that included 460 patients who were diagnosed with treatment-naïve neovascular AMD and PCV and were initially treated with either ranibizumab or aflibercept. The patients were divided into two groups: the ranibizumab group (*n* = 96) and the aflibercept group (*n* = 324). The patients’ characteristics and the proportion of the subtypes of macular neovascularization (MNV) were compared between the two groups. RESULTS: Patients in the ranibizumab group were significantly older (mean 74.3 ± 8.4 years) than those in the aflibercept group (mean 70.4 ± 8.8 years; *p* < 0.001). In the ranibizumab group, the proportions of type 1 or 2 MNV, type 3 MNV, and PCV were 50.0%, 27.1%, and 22.9%, respectively. In the aflibercept group, the proportions were 35.2%, 6.8%, and 58.0%, respectively. There was a significant difference in the proportion of MNV subtypes between the ranibizumab and aflibercept groups (*p* < 0.001). Ranibizumab was used in 54.2% of patients with type 3 MNVs. However, in patients with PCV, aflibercept was used in 89.5% of patients. CONCLUSIONS: Ranibizumab was preferred as an initial treatment agent in older patients and those with type 3 MNV, whereas aflibercept was highly preferred in patients with PCV. The different characteristics and efficacy of the two agents may have partially contributed to this trend.

## 1. Introduction

Neovascular age-related macular degeneration (AMD) is a type of late AMD which can lead to visual loss. With the increasing global prevalence of AMD [1], the importance of neovascular AMD treatment is expected to increase in the future. Polypoidal choroidal vasculopathy (PCV), also known as aneurysmal type 1 neovascularization [2], is a peculiar type of macular neovascularization (MNV) prevalent in the Asian population [3]. Although there are differences in the characteristics between neovascular AMD and PCV, these two MNVs usually respond well to anti-vascular endothelial growth factor (VEGF) therapy.

Ranibizumab and aflibercept are widely used anti-VEGF agents, approved by the Food and Drug Administration (FDA), for the treatment of neovascular AMD and PCV. Although these two agents have different characteristics [4], there is no gold standard for selecting the agent for the initial treatment of neovascular AMD or PCV, and the selection is generally the personal discretion of each treating physician. In previous real-world studies, some differences in the usage of these two agents have been noted [5,6]. However, the reasons for these differences have not yet been fully investigated.

In selecting anti-VEGF agents, physicians may have their own guidelines based on personal experience and knowledge, resulting in some differences in the selection patterns among the physicians. Identifying the selection patterns of other physicians and investigating the potential reasons for their development would be of value because it may provide useful information in determining one’s own treatment strategy.

In the present study, the trend of selecting ranibizumab and aflibercept for the initial treatment of neovascular AMD and PCV was investigated. We particularly focused on the preference of a particular anti-VEGF agent for certain conditions and discussed the potential reasons for this preference.

## 2. Materials and Methods

This retrospective observational study was conducted in a single center (Kim’s Eye Hospital, Seoul, Korea). The study was approved by the Institutional Review Board of Kim’s Eye Hospital (#2021-02-003) and was conducted in accordance with the tenets of the Declaration of Helsinki.

### 2.1. Patients, Examinations, and Treatment

This study included patients who were diagnosed with treatment-naïve neovascular AMD and PCV between January 2020 and December 2020 and were initially treated with ranibizumab or aflibercept. The exclusion criteria were as follows: (1) patients without indocyanine green angiography (ICGA) results and (2) patients who underwent ICGA examination, but the subtypes of MNV could not be accurately classified due to media opacity or extensive subretinal hemorrhage. When both eyes met the eligibility criteria, the eye with the prior symptom onset was included in the study.

At diagnosis, all patients underwent slit-lamp biomicroscopy, fundus examination using a 90D lens, and measurement of best-corrected visual acuity (BCVA). Fluorescein angiography, IGCA, and optical coherence tomography (OCT) images were obtained using Spectralis HRA + OCT^®^ (Heidelberg Engineering GmbH, Heidelberg, Germany). Fundus photographs were also captured.

After diagnosis, the patients initially received three monthly loading injections of either ranibizumab (0.5 mg/0.05 mL of Lucentis^®^; Genentech Inc., San Francisco, CA, USA) or aflibercept (2.0 mg/0.05 mL of Eylea^®^; Regeneron Pharmaceuticals, Tarrytown, NY, USA). At our institution, 19 ophthalmologists are specialized in the treatment of retinal diseases. Since there are no common guidelines for the selection of anti-VEGF agents, the selection was solely based on the personal discretion of each treating physician. Generally, retreatment after three monthly loading injections was performed on an as-needed basis. The treat-and-extend (TAE) regimen was used for selected patients at the discretion of treating physicians.

### 2.2. Classification of Subtypes of MNV

**Figure 1:**
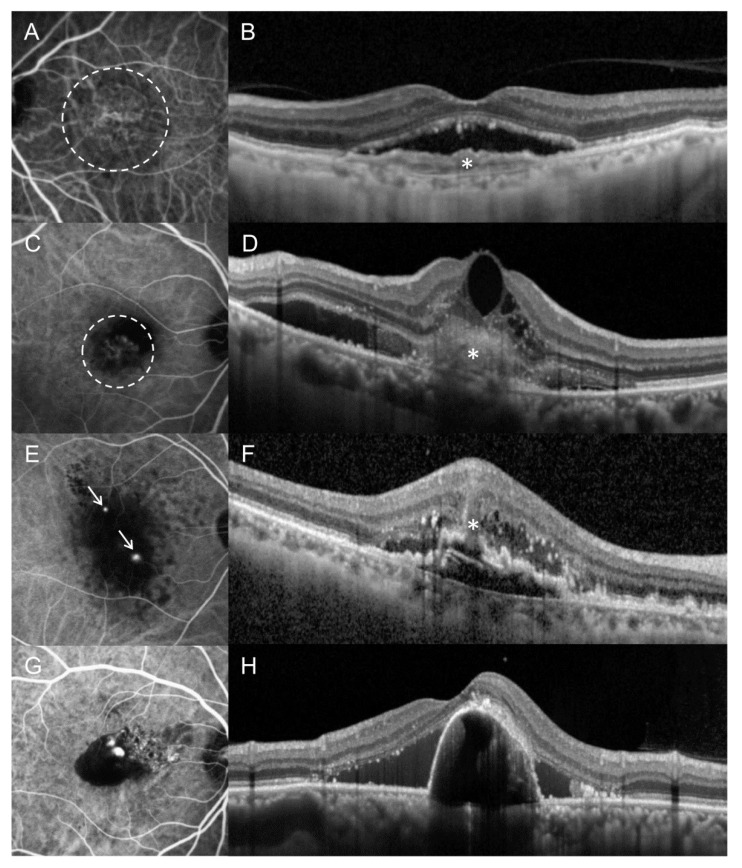
Representative indocyanine green angiography (ICGA) (**A**,**C**,**E**,**G**) and optical coherence tomography (OCT) (**B**,**D**,**F**,**H**) images showing representative cases of type 1 macular neovascularization (MNV) (**A**,**B**), type 2 MNV (**C**,**D**), type 3 MNV (**E**,**F**), and polypoidal choroidal vasculopathy (PCV) (**G**,**H**). In type 1 MNV, a neovascular complex, observed on ICGA (**A**, dashed circle), is confined to the sub-retinal pigment epithelium (**B**, asterisk). In type 2 MNV, a neovascular complex was noted on ICGA (**C**, dashed circle), accompanied with subretinal hyperreflective materials on OCT (**D**, asterisk). In type 3 MNV, focal hyperfluorescent lesions are observed on ICGA (**E**, arrows). On OCT, an intraretinal lesion with medium to high reflectivity was noted, accompanied with intraretinal edema and disruption of retinal pigment epithelial layer (**F**, asterisk). In PCV, polypoidal lesions, observed on ICGA (**G)**, are accompanied by prominent serous pigment epithelial detachment and subretinal fluid, observed on OCT (**H**).

**Figure 2:**
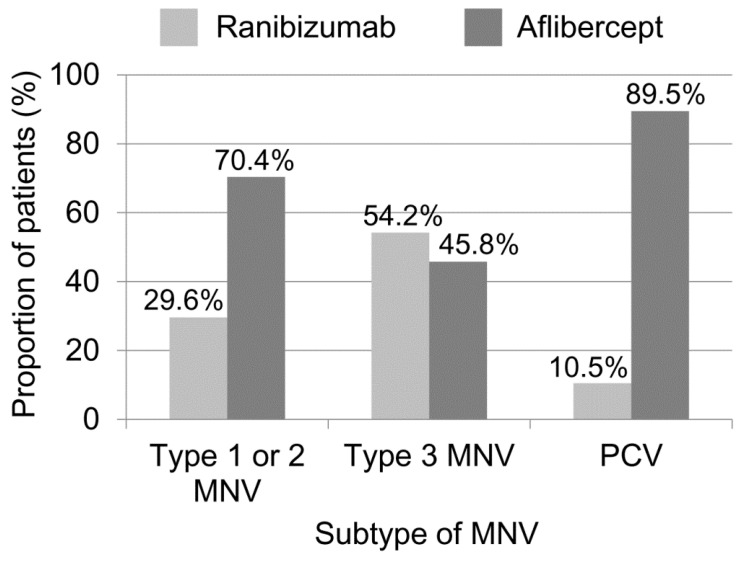
Proportion of patients who received ranibizumab or aflibercept as an initial treatment, according to the subtypes of macular neovascularization (MNV).

The MNVs were classified into three groups based on the ICGA and OCT findings (Figure 1). Eyes exhibiting polypoidal lesions with or without branching vascular networks on ICGA were classified as PCV (Figure 1G,H). Eyes exhibiting intraretinal lesions with or without focal disruption of the retinal pigment epithelium (RPE) on OCT, accompanied by focal hyperfluorescence on angiography [7], were classified as type 3 MNVs (Figure 1E,F). Eyes without PCV or type 3 MNV features were classified as type 1 (Figure 1A,B) or 2 MNV (Figure 1C,D).

### 2.3. Result Analyses

Patients who were initially treated with ranibizumab were included in the ranibizumab group, and those treated with aflibercept were included in the aflibercept group. The following baseline characteristics were compared between the two groups: age, sex, diabetes mellitus, hypertension, lens status, type of MNV, and BCVA. In addition, the patients’ age and sex were compared among those with type 1 or 2 MNV, type 3 MNV, and PCV. In type 1 or 2 MNV, patients’ age was compared between the ranibizumab and aflibercept groups. BCVAs were converted to logarithm of the minimum angle of resolution (logMAR) values for analysis. “Counting fingers” and “hand motion” visual acuities were converted to logMAR values of 2 and 3, respectively [8].

### 2.4. Statistical Analyses

The data are presented as mean ± standard deviation or as a number (percentage), wherever applicable. Statistical analyses were performed using a commercially available software package (SPSS version 12.0; IBM Corporation, Armonk, NY, USA). The Shapiro–Wilk test was used to test the normality of the data. Comparisons between the ranibizumab and aflibercept groups were performed using the independent samples *t*-test, Mann–Whitney U test, or chi-squared test. Comparisons among the different subtypes of MNV were performed using the one-way analysis of variance with Tukey’s test and chi-squared test. Statistical significance was set at *p* < 0.05.

## 3. Results

A total of 460 patients were diagnosed with treatment-naïve neovascular AMD and received loading injections of ranibizumab or aflibercept. Among these, 40 patients were excluded for the following reasons: (1) lack of ICGA results or subtypes of MNV could not be accurately classified (38 patients) and (2) enrolled in a clinical trial (2 patients). Finally, 420 patients (420 eyes; 255 men and 165 women) were included in the analysis (Table 1). The mean age was 71.3 ± 8.9 years. Of these, 162 (38.6%) were classified as type 1 or 2 MNV, 48 (11.4%) as type 3 MNV, and 210 (50.0%) as PCV. The mean logMAR BCVA was 0.62 ± 0.53 (Snellen equivalent = 20/83).

Ranibizumab was selected in 96 patients (22.9%), whereas aflibercept was selected in 324 patients (77.1%). Differences in characteristics between the ranibizumab and aflibercept groups are summarized in Table 2. Patients in the ranibizumab group were significantly older (mean 74.3 ± 8.4 years) than those in the aflibercept group (mean 70.4 ± 8.8 years; *p* < 0.001). In addition, the proportion of women was significantly higher in the ranibizumab group (61.5%) than in the aflibercept group (32.7%; *p* < 0.001). There was a significant difference in the proportion of MNV subtypes between the ranibizumab and aflibercept groups (*p* < 0.001). In the ranibizumab group, the proportion of type 3 MNVs (27.1%) was relatively higher than that in the aflibercept group (6.8%), but the proportion of PCV (22.9%) was relatively lower than that in the aflibercept group (58.0%). In patients with type 1 or 2 MNV, ranibizumab was used in 48 patients (29.6%) and aflibercept was used in 114 (70.4%; Figure 2). In patients with type 3 MNV, ranibizumab and aflibercept were used in 26 (54.2%) and 22 (45.8%) patients, respectively. In patients with PCV, ranibizumab and aflibercept were used in 22 (10.5%) and 188 (89.5%) patients, respectively.

Comparisons among patients with the three subtypes of MNV revealed a significant difference in age (*p* < 0.001) and sex (*p* < 0.001; Table 3). The patients with type 3 MNV were significantly older (mean 76.6 ± 8.9 years) than those with type 1 or 2 MNV (mean 72.5 ± 8.9 years, *p* = 0.010) and PCV (mean 69.2 ± 8.6 years, *p* < 0.001). Patients with type 1 or 2 MNV were significantly older than those with PCV (*p* = 0.001). The proportion of women was highest among patients with type 3 MNV (87.5%), followed by those with type 1 or 2 MNV (43.2%) and PCV (25.2%). In type 1 or 2 MNV, the mean age was 75.3 ± 8.5 years in the ranibizumab group and 71.3 ± 8.9 years in the aflibercept group. Patients in the ranibizumab group were significantly older than those in the aflibercept group (*p* = 0.009).

Among the 38 patients who were excluded from the main analyses due to lack of ICGA results, ranibizumab was selected as the initial treatment in 13 patients (34.2%) and aflibercept was selected in the remaining 25 patients (65.8%).

## 4. Discussion

In the present study, there was a notable trend in the selection of an anti-VEGF agent for the initial treatment of neovascular AMD and PCV. Ranibizumab was preferred for older patients and those with type 3 MNV. In contrast, aflibercept was highly preferred for PCV treatment. In patients without ICGA results, ranibizumab was used in approximately one-third of patients and aflibercept was used in approximately two-thirds of patients. Interestingly, this proportion was similar to that noted in type 1 or 2 MNV (ranibizumab: 29.6%; aflibercept: 70.4%). Thus, the decision of the treating physicians would contribute to the marked difference in the usage of anti-VEGF agents between patients with type 3 MNV and PCV. The following are our postulations explaining why certain anti-VEGF agents were preferred under certain conditions.

PCV is a subtype of MNV that is prevalent in Asians [3]. It has characteristics distinct from those of typical neovascular AMD, such as the presence of typical polypoidal lesions on ICGA [9], relatively benign natural course [10], thick choroid [11], and occurrence at a relatively young age [12]. Despite these differences, anti-VEGF agents, which were originally developed to treat neovascular AMD, are also effective in treating PCV [3]. However, previous studies have shown that eyes treated with aflibercept generally show a higher rate of resolution of polypoidal lesions and a greater decrease in retinal thickness than those treated with ranibizumab [13,14]. In addition, switching from ranibizumab to aflibercept may be beneficial in refractory PCV [15,16]. Although the exact reason for the difference in efficacy is unclear, several investigators have postulated that it could be due to the difference in the effect of the two agents on the choroid [17]. Recently, the efficacy of aflibercept monotherapy was found to be similar to that of aflibercept therapy with rescue photodynamic therapy [18], suggesting a good efficacy of aflibercept in PCV. In the present study, aflibercept was highly preferred for the initial treatment of PCV cases. Hence, we postulate that the relatively superior outcomes of aflibercept over ranibizumab, as shown in previous studies, have influenced this result. In fact, aflibercept was the most frequently used FDA-approved anti-VEGF agent to treat PCV in the Asia-Pacific region [5]. This result, along with the results of the present study, may show a tendency to prefer aflibercept in PCV treatment.

Type 3 MNV is a subtype of MNV characterized by intraretinal neovascularization [19,20]. In the present study, ranibizumab was selected in more than half of the patients with type 3 MNV. This is a notable finding because aflibercept was preferred in the other subtypes of MNV. We postulate two issues that may have influenced this trend. The first issue is RPE atrophy. It is well known that patients with type 3 MNV are at a high risk of developing RPE atrophy [21,22]. Eyes with type 3 MNV usually exhibit a very thin choroid [23], and thinner choroid in eyes with type 3 MNV is considered to be associated with a higher risk of RPE atrophy [24]. Thus, choroidal thinning is an important issue in the treatment of type 3 MNV. In general, aflibercept induces a higher degree of choroidal thinning than does ranibizumab [17]. Therefore, it is important to determine whether aflibercept can facilitate RPE atrophy. In a recent clinical trial, the incidence of RPE atrophy was found to be similar between eyes treated with aflibercept and ranibizumab [25]. However, several investigators have also demonstrated that the incidence of RPE atrophy is slightly higher in type 3 MNVs treated with aflibercept than in those treated with ranibizumab [26]. Although there is no firm evidence suggesting the influence of aflibercept on RPE atrophy, the possibility of an influence on the selection of anti-VEGF agents in type 3 MNVs cannot be completely ignored.

The second issue concerns the different systemic effects of ranibizumab and aflibercept. It is well known that a part of intravitreally injected anti-VEGF agent enters the systemic circulation, leading to a decrease in systemic VEGF levels [27]. In general, this side effect of anti-VEGF therapy may not induce clinically significant complications, such as myocardial infarction or stroke [28]. However, some concerns have been raised regarding the association of prolonged, frequent anti-VEGF treatment with a higher incidence of cerebrovascular accidents [29]. An important issue is that there is a difference in the systemic effects among different anti-VEGF agents. More specifically, aflibercept induced a more profound decrease in systemic VEGF levels than did ranibizumab [27]. Previously, Avery analyzed the VIEW study data and pointed out that the incidence of cerebral vascular events was relatively higher in older patients treated with aflibercept than in those treated with ranibizumab [30]. Beaumont also raised a similar issue [31]. Recently, Barthelmes et al. investigated trends in the use of ranibizumab and aflibercept and found that patients treated with ranibizumab were significantly older than those treated with aflibercept [6]. The authors postulated that this trend was partially due to the previously suggested concern regarding the systemic impact of aflibercept in older patients [6]. Hence, it is important to note that type 3 MNVs usually develop in older subjects [12]. In the present study, patients with type 3 MNVs were significantly older than those with the other subtypes of MNV. Thus, we postulate that the higher usage of ranibizumab for type 3 MNVs in the present study was partially influenced by concerns regarding systemic side effects in older patients. When analyzing within the type 1 or 2 MNV group, patients treated with ranibizumab were older than those treated with aflibercept. This result may support our postulation.

In our institution, the TAE regimen is implemented after initial loading injections at the discretion of treating physicians. Recently, Ohji et al. reviewed the results of randomized controlled clinical trials of neovascular AMD and compared the outcomes of the TAE regimen with ranibizumab with those of the TAE regimen with aflibercept [32]. Consequently, they found that compared with the TAE regimen with ranibizumab, the TAE regimen with aflibercept improved long-term visual acuity with fewer injections. The authors concluded that the TAE regimen with aflibercept can serve as the optimal therapy for neovascular AMD [32]. It is possible that the results of these previous studies have an influence on the relatively higher preference for aflibercept when planning TAE-based retreatment.

In the present study, ranibizumab was more preferred in women than in men. To date, there is no evidence of a difference in the efficacy of different anti-VEGF agents between men and women. Thus, sex-related differences in the usage of agents may be a secondary finding. The majority of our patients with type 3 MNV were women, whereas the majority of patients with PCV were men. Since ranibizumab was preferred for type 3 MNV and aflibercept was preferred for PCV, this distinct trend in the use of anti-VEGF agents might have caused the sex-related difference. Aflibercept was more preferred in phakic patients than in pseudophakic patients. There is no evidence suggesting any difference in the efficacy of anti-VEGF agents according to lens status. We believe that the higher proportion of phakic patients in the aflibercept group is mainly due to the relatively younger age of the patients in the aflibercept group than in the ranibizumab group.

It is well known that the price of anti-VEGF agents varies across countries [33,34]. In addition, the price can differ according to health insurance plans [33]. In South Korea, the price of anti-VEGF agents is strictly controlled by the government. During the study period, the prices of ranibizumab and aflibercept were similar (cost of ranibizumab = 828,166 Korean won (approximately 720 US dollars); cost of aflibercept = 783,920–793,360 Korean won (approximately 682–689 US dollars)). In addition, neovascular AMD is considered an “intractable disorder” in the Korean national insurance system. Therefore, the Korean national insurance system has a special medical expense support system that covers 90% of the expenses of ranibizumab and aflibercept treatments for neovascular AMD. The financial burden on the patient is only 10% of the total drug price. Hence, we postulate that difference in the prices of ranibizumab and aflibercept may have had a limited influence on the study results.

The purpose of the present study was not to address the question “Which anti-VEGF agent should be selected in a specific condition?”; both ranibizumab and aflibercept are widely used, safe, and effective treatments for neovascular AMD and PCV. We merely presented the trend of anti-VEGF usage and attempted to provide potential explanations based on the known pros and cons of each anti-VEGF agent.

In addition to its retrospective nature, the present study has some limitations. This study was performed at a single center, and all the included patients were Korean. Since PCV is particularly prevalent in Asian populations [35], the results of the present study may not be valid in other ethnic groups. In addition, the selection of anti-VEGF agents can be influenced by the price of the agent as well as the reimbursement policy of the national health insurance system or other insurance providers. Hence, the results of the present study may not be directly applicable to other countries. Lastly, bevacizumab usage was not evaluated. In South Korea, the use of bevacizumab is generally determined by financial rather than scientific reasons. Thus, patients initially treated with bevacizumab were not included in the study to avoid bias.

In summary, we evaluated the trend of selecting ranibizumab and aflibercept as initial treatments for neovascular AMD or PCV. Ranibizumab was preferred in older patients and those with type 3 MNV, whereas aflibercept was preferred in those with PCV. The influence of the introduction of new anti-VEGF agents, such as brolucizumab, on this trend merits further investigation. In addition, continuous research along with expert discussions will be required to determine the appropriate anti-VEGF agent for a specific condition.

## Figures and Tables

**Table 1:** Patient characteristics (*n* = 420).

Characteristics	Value
Age (years)	71.3 ± 8.9
Sex (male: female)	255 (60.7%): 165 (39.3%)
Diabetes mellitus	89 (21.2%)
Hypertension	200 (47.6%)
Type of MNV	
Type 1 or 2 MNV	162 (38.6%)
Type 3 MNV	48 (11.4%)
Polypoidal choroidal vasculopathy	210 (50.0%)
Lens status	
Phakic	287 (68.3%)
Pseudophakic	133 (31.7%)
Best-corrected visual acuity (logMAR)	0.62 ± 0.53
Type of anti-VEGF agent	
Ranibizumab	96 (22.9%)
Aflibercept	324 (77.1%)

Data are presented as mean ± standard deviation or number (%), when applicable. VEGF: vascular endothelial growth factor; MNV: macular neovascularization; logMAR: logarithm of the minimum angle of resolution.

**Table 2:** Comparison of the parameters between the regression group and non-regression group.

Parameters	Ranibizumab Group (*n* = 96)	Aflibercept Group (*n* = 324)	*p* Value
Age (years)	74.3 ± 8.4	70.4 ± 8.8	<0.001 *
Sex (men: women)	37 (38.5%): 59 (61.5%)	218 (67.3%): 106 (32.7%)	<0.001 ^†^
Diabetes mellitus	22 (22.9%)	67 (20.7%)	0.637 ^†^
Hypertension	48 (50.0%)	152 (46.9%)	0.595 ^†^
Type of MNV			<0.001 ^†^
Type 1 or 2 MNV	48 (50.0%)	114 (35.2%)	
Type 3 MNV	26 (27.1%)	22 (6.8%)	
Polypoidal choroidal vasculopathy	22 (22.9%)	188 (58.0%)	
Lens status			0.016 ^†^
Phakic	56 (58.3%)	231 (71.3%)	
Pseudophakic	40 (41.7%)	93 (28.7%)	
Best-corrected visual acuity (logMAR)	0.69 ± 0.52	0.60 ± 0.53	0.055 ^‡^

Data are presented as mean ± standard deviation or number (%), when applicable. MNV: macular neovascularization; logMAR: logarithm of the minimum angle of resolution. * Statistical analysis using the independent samples *t*-test. ^†^ Statistical analysis using the chi-squared test. ^‡^ Statistical analysis using the Mann–Whitney U test.

**Table 3:** Comparisons of characteristics among different types of macular neovascularization.

Characteristics	Type 1 or 2 MNV (*n* = 162)	Type 3 MNV (*n* = 48)	PCV (*n* = 210)	*p* Value
Age (years)	72.5 ± 8.9	76.6 ± 6.6	69.2 ± 8.6	<0.001 *
Sex (male: female)	92 (56.8%): 70 (43.2%)	6 (12.5%): 42 (87.5%)	157 (74.8%): 53 (25.2%)	<0.001 ^†^

Data are presented as mean ± standard deviation or number (%), when applicable. MNV: macular neovascularization; PCV: polypoidal choroidal vasculopathy. * Statistical analysis using one-way analysis of variances. ^†^ Statistical analysis using the chi-squared test.

## Data Availability

The data presented in this study are available on reasonable request from the corresponding author.
